# External Validation of PathFx 3.0 Model on a Single-Centre Portuguese Population

**DOI:** 10.7759/cureus.104218

**Published:** 2026-02-25

**Authors:** João Rosado, Vera Vaz, Sofia Miguel, Renata Vaz, Jorge Rebola, João Nobrega, Pedro Rasteiro, Joana Correia, Carlos Pedrosa, José Portela

**Affiliations:** 1 Orthopaedics, Unidade Local de Saúde de São José, Lisbon, PRT; 2 Orthopaedics, Unidade Local de Saúde Amadora/Sintra, Lisbon, PRT

**Keywords:** bone tumors, internal fixation, orthopaedic oncology and arthroplasty, skeletal metastasis, survival analysis

## Abstract

Purpose

PathFx 3.0 (Jonathan A. Forsberg, Memorial Sloan Kettering Cancer Center, New York, NY, USA; Rikard Wedin, Karolinska Institutet, Stockholm, Sweden) is a prognostic model designed to predict survival in patients with bone metastatic disease. While it has been widely validated, concerns remain regarding its application across different healthcare systems. This study aimed to externally validate the performance of PathFx 3.0 in a Portuguese cohort.

Methods

A retrospective cohort of patients who underwent surgery for skeletal metastases between January 2018 and April 2023 was analysed. Survival probabilities at one, three, six, 12 and 24 months were generated for each patient. Performance was assessed using the area under the receiver operating characteristic curve (AUC-ROC) with 95% confidence intervals, Brier scores and calibration.

Results

A total of 49 patients were included, with a median follow-up of 19 months (interquartile range (IQR) 5-35). Observed survival was 89.8% at one month, 81.6% at three months, 69.4% at six months, 65.3% at 12 months and 42.9% at 24 months. Discrimination was poor at one month (area under the curve (AUC) 0.495) but improved in subsequent time periods, with AUC values of 0.760 at three months, 0.773 at six months, 0.791 at 12 months and 0.752 at 24 months. Brier scores ranged from 0.172 to 0.246. Calibration analysis showed good agreement between predicted and observed outcomes, except in the early post-operative period.

Conclusion

PathFx 3.0 demonstrated good performance in the studied population. While early survival prediction remains limited, the model provides valuable prognostic support for operative decision-making in patients with metastatic bone disease in later time periods. Continued external validation in larger, multicentric Portuguese cohorts is warranted.

## Introduction

Surgical treatment for patients with bone metastasis has been under constant evolution over the last few decades. Restoring function, reducing pain and improving the quality of life are the main goals of our approach as orthopaedic surgeons. However, as new oncologic treatments are developing and patient survival keeps expanding, focus has shifted to account for implant longevity in our treatment choice [1,2]. An individualised treatment strategy is currently the most supported rationale among experts [3-5], stating two major corollaries for the orthopaedic approach: first, the implant should outlive the patient while providing pain relief and restoring function [6,7]; second, patients should not be subjected to major, high-risk reconstructions if their prognosis deems it unlikely that they will benefit from it.

To consistently apply such principles, predicting patient survival has undergone major research across specialist centres. What was once based solely on the medical team’s somewhat subjective survival estimation, which tends to be rather optimistic [8], is now integrated with machine-learning or AI-based models to complement physician assessment. Many of these prognostic models were developed, updated and externally validated over the past 15 years [9-11], mainly using objective parameters to establish prognosis and aid in our clinical decisions.

Developed in 2011, PathFx (Jonathan A. Forsberg, Memorial Sloan Kettering Cancer Center, New York, NY, USA; Rikard Wedin, Karolinska Institutet, Stockholm, Sweden) has been one of the most tested models. It is based on previously identified survival predictors [12] to generate survival probabilities for five different time periods (one, three, six, 12 and 24 months). Since it was developed through a Bayesian Belief Network system, it can provide those predictions across multiple oncologic subtypes. Furthermore, it is able to maintain its accuracy even in the event of missing data [13]. It has a user-friendly interface, and it is designed for use in a daily practice setting.

Although PathFx has been subjected to multiple updates (assuring its accuracy as new oncologic treatments emerge) and validated across different populations [14-17], there is still concern about the applicability of a clinical decision tool across different populations, as health care access, treatment availability or protocols can vary, even among European Union countries.

The purpose of this study was therefore to validate this prognostic model in our patient population, to ensure that its predictions can guide our clinical decisions.

## Materials and methods

We conducted a retrospective, single-centre external validation study of the PathFx 3.0 survival prediction model in surgically treated patients with skeletal metastases. Study design and statistical analysis were modelled after Riley et al.’s British Medical Journal series, “Evaluation of Clinical Prediction Models (part 2): how to undertake an external validation study” [18].

This study was conducted in a tertiary public hospital within the Portuguese National Health Service. We retrospectively screened consecutive patients undergoing index surgery for skeletal metastases at our institution from 2018 to April 2023, using the department’s database; only the first eligible procedure per patient was considered. Patients were excluded if essential predictor data required to compute the model were missing, or if vital status and/or date of last follow-up could not be ascertained. Vital status and follow-up were obtained from the electronic medical record and the national registry, with patients censored at the last documented follow-up. 

Baseline demographic, clinical, oncological and laboratory variables required to compute PathFx predictions were obtained from electronic medical records - oncological diagnosis (classified according to Forsberg et al. [14]); demographics; Eastern Cooperative Oncological Group Performance Status [19] (or similar, e.g. Karnofsky Performance Status); number of bone metastasis present; haemoglobin concentration; absolute lymphocyte count; presence of visceral, brain or lymph node metastasis and status. Senior oncologist/surgeon survival estimation was not considered for model input.

PathFx 3.0 provides predicted probabilities of survival at multiple time horizons. For each patient, predicted survival probabilities at one, three, six, 12 and 24 months were extracted and analysed as continuous predicted risks. No model updating or refitting was performed prior to validation.

Overall survival was defined as the time from the date of surgery to death from any cause. Patients were censored at the last known follow-up. For each time horizon (one, three, six, 12 and 24 months), a binary outcome was defined indicating survival beyond that time point (alive vs. deceased at or before the horizon).

All statistical analyses were performed using R (version 4.5.0; R Foundation for Statistical Computing, Vienna, Austria). Survival functions were estimated using the Kaplan-Meier method with the survival package.

Model performance was evaluated across all horizons using complementary metrics of discrimination, calibration and overall accuracy, consistent with external validation recommendations [18]. Discrimination was assessed using the area under the receiver operating characteristic curve (AUC-ROC) with 95% confidence intervals calculated using DeLong’s method (pROC package, Xavier Robin, University of Basel Biozentrum, Basel, Switzerland). Overall accuracy was assessed using the Brier score (rms package, Frank E. Harrell Jr., Vanderbilt University School of Medicine, Nashville, TN).

Calibration was assessed using logistic recalibration, estimating the calibration intercept (calibration-in-the-large) and calibration slope by regressing the observed binary outcome on the logit of the predicted survival probability at each horizon. Graphical calibration was examined using locally estimated scatterplot smoothing (LOESS)-smoothed calibration curves; due to the modest sample size, curves were constructed using quintiles of predicted risk, plotting mean predicted probability versus observed survival within each stratum and overlaying a smooth trend.

## Results

A total of 56 patients who underwent surgical treatment for skeletal metastases were identified. Seven were excluded due to missing Eastern Cooperative Oncology Group (ECOG) performance status required to compute PathFx predictions, leaving 49 patients for external validation (Table 1).

**Table 1:** Cohort assembly and exclusions The unit of analysis was the patient. If multiple eligible procedures occurred in the same patient, the first eligible procedure was considered the index case. ECOG, Eastern Cooperative Oncology Group; IQR, interquartile range

Step	n	Details
Patients screened for eligibility (surgery for skeletal metastases)	56	Identified from the department's database from January 2018 to April 2023
Excluded	7	Missing ECOG/performance status required to compute PathFx predictions
Included in the external validation cohort	49	Complete case for required model inputs
Follow-up (months), median (IQR)/mean	19 (5-35)/21.5	Postoperative follow-up

**Figure 1:**
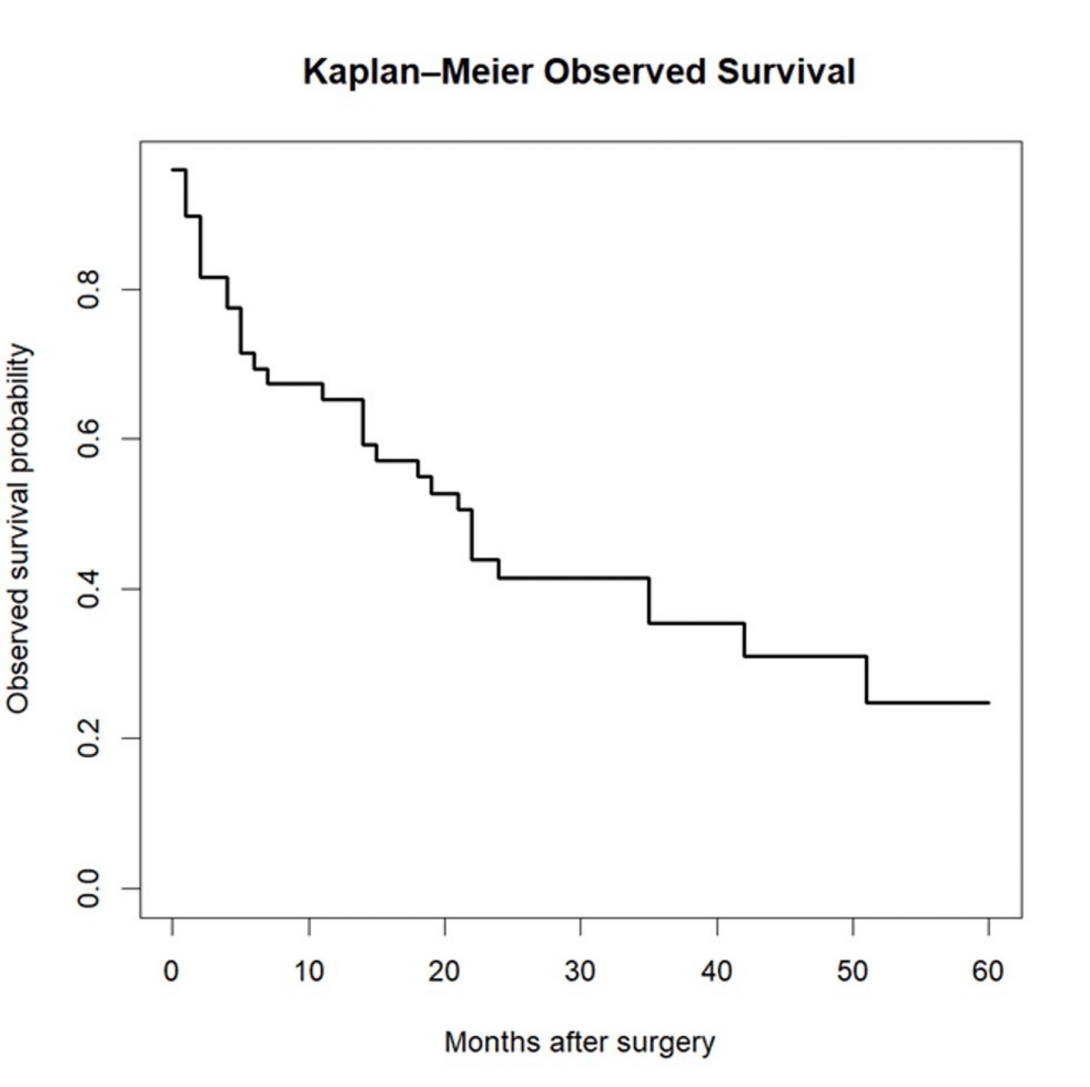
Kaplan-Meier observed survival

Median postoperative follow-up was 19 months (interquartile range (IQR) 5-35), with a mean follow-up of 21.5 months. Baseline cohort characteristics are summarised in Table 2. Observed survival remained high in the early postoperative period, with 89.8% alive at one month, 81.6% at three months, 69.4% at six months, 65.3% at 12 months and 42.9% at 24 months. Kaplan-Meier curves are displayed in Figure 1.

**Table 2:** Patient characteristics Diagnosis group 1 - lung, hepatocellular, gastric carcinoma or melanoma. Diagnosis group 2 - sarcoma and other carcinomas not in groups 1 or 3. Diagnosis group 3: breast (except triple negative), thyroid (follicular/papillary) or prostate adenocarcinoma. Eastern Cooperative Oncology Group (ECOG) Performance Status: 0 - fully active; 1 - restricted in strenuous activity; 2 - ambulatory and capable of all self-care; 3 - capable of only limited self-care; 4 - completely disabled.

Age (years)	
Mean (SD)	65.78 (13.64)
Haemoglobin (g/dL)	
Mean (SD)	11.82 (2.03)
Lymphocyte count (cells/µL)	
Mean (SD)	1486.73 (625.38)
Sex	
Female	23 (46.9%)
Male	26 (53.1%)
Diagnosis group [14]	
1	9 (18.4%)
2	10 (20.4%)
3	30 (61.2%)
Fracture status	
Pathological	24 (49.0%)
Impending	25 (51.0%)
ECOG Performance Status [19]	
0	24 (49.0%)
1	15 (30.6%)
2	5 (10.2%)
3	5 (10.2%)
4	0 (0%)
Skeletal metastases	
Solitary	18 (36.7%)
Multiple	31 (63.3%)
Visceral/brain metastases	
No	28 (57.1%)
Yes	21 (42.9%)
Lymph node metastases	
No	24 (49.0%)
Yes	25 (51.0%)

Discrimination varied across time horizons. At one month, discrimination was poor (area under the curve (AUC) 0.495; 95% CI 0.14-0.87), consistent with performance no better than chance and likely influenced by the small number of early events. From three months onwards, the model demonstrated moderate-to-good discriminatory capacity, with AUC values between 0.760 and 0.791 [20] at three to 12 months and 0.752 at 24 months (Table 3). However, precision was limited, with relatively wide 95% confidence intervals reflecting the modest sample size.

**Table 3:** Model overall performance AUC-ROC values are presented with DeLong 95% confidence intervals in parentheses. The Brier score represents the mean squared error of predicted probabilities (lower values indicate better overall accuracy). Calibration intercept: ideal value = 0 (positive values indicate systematic under-prediction of survival). Calibration slope: ideal value = 1 (values < 1 indicate overly extreme predictions; values > 1 indicate under-dispersion of predicted probabilities). AUC-ROC, area under the receiver operating characteristic curve; CI, confidence interval

	AUC-ROC (95% CI)	Brier Score	Intercept	Slope
One month	0.495 (0.14-0.87)	0.094	2.463	-0.102
Three months	0.760 (0.58-0.94)	0.172	1.236	1.152
Six months	0.773 (0.62-0.92)	0.231	1.280	1.081
12 months	0.791 (0.66-0.92)	0.246	1.524	1.346
24 months	0.752 (0.61-0.90)	0.218	0.772	0.966

Brier scores, reflecting overall accuracy, were lowest at one month (0.094) and highest at 12 months (0.246) (Table 3). The very low one-month Brier is expected in a setting with high short-term survival and should be interpreted in the context of limited early events. Across clinically informative horizons (three to 24 months), Brier scores ranged from 0.172 to 0.246, indicating acceptable overall performance. Calibration intercepts were consistently positive, indicating systematic underestimation of survival probabilities.

**Figure 2:**
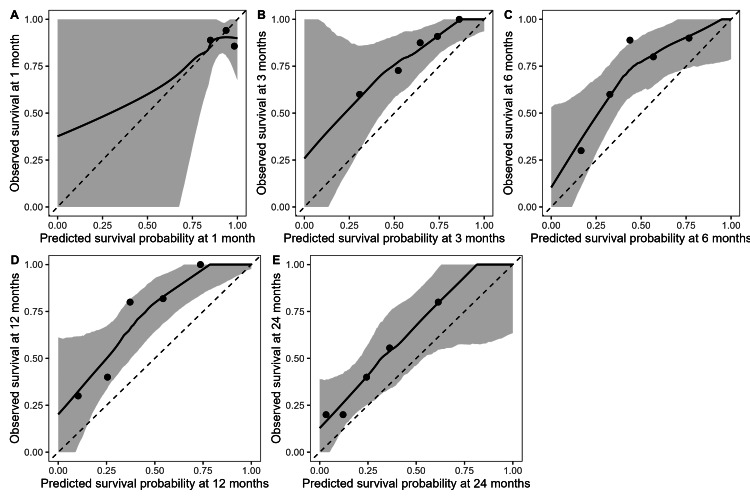
Calibration curves for predicted survival Figures A-E correspond to one-, three-, six-, 12- and 24-month survival predictions, respectively. The solid black line represents the locally estimated scatterplot smoothing (LOESS)-smoothed calibration curve, the grey band indicates the 95% bootstrap confidence interval, and the dashed line represents perfect calibration. Points represent quintile-based mean predicted and observed survival.

Calibration analysis showed substantial underestimation of survival at one month, as reflected by a high calibration intercept (2.463) and a slope approaching zero. Similar to the AUC-ROC and Brier scores, calibration improved significantly for later horizons. Intercepts ranged from 0.77 to 1.52, reflecting mild survival underestimation. Meanwhile, slopes were close to one, demonstrating an accurate spread of predicted probabilities. These findings are illustrated in Figure 2.

Smoothed curves (Figure 2) for three, six, 12 and 24 months show adequate calibration, generally tracking the ideal 45-degree line. Confidence bands widened at higher predicted probabilities, consistent with smaller subgroup samples. Quintile-based plots revealed that the model performed especially well in the midrange of predicted probabilities, with slight underestimation at the upper extremes.

## Discussion

These findings support the use of PathFx 3.0 as a useful adjunct to aid our clinical decision in our patient population when estimating survival beyond the immediate postoperative phase, particularly at three, six and 12 months.

In this surgically treated cohort, PathFx 3.0 showed moderate-to-good discrimination from three months onwards, consistent with previously reported external validations [11,15-17,21-23]. However, estimates were less precise due to the modest sample size, and calibration metrics indicated systematic underestimation of survival, albeit with improved calibration at later timepoints. Discrimination at one month was not informative in this dataset, likely because early mortality was low in this selected surgical population, as also described in prior studies [15,23]. Taken together, the model’s performance at the most clinically relevant postoperative horizons - particularly six and 12 months [24-26] - supports its use as a decision-support tool alongside clinical judgement.

This study is not without its limitations. Firstly, our small cohort limits the grade of our recommendation to use PathFx. Due to the lack of pathological fracture treatment centralisation in our region, a multicentric study would be appropriate to increase the robustness of our validation. Secondly, the model’s poor performance at one month limits its use in the clinically important setting of deciding between surgical stabilisation or palliative non-invasive treatment. Including patients in whom non-surgical treatment was decided would allow us to properly test PathFx’s performance in the early post-operative period. Thirdly, clinical utility analysis is lacking. It was our opinion that we could not undertake such a step without a minimum 24-month follow-up for all patients, since we would not account for osteosynthesis failures in the long survivors [24,27].

## Conclusions

Despite its limitations, it is our opinion that this data supports the use of PathFx as a complementary tool to guide the choice of treatment in our population. However, we must remain cautious of its application in patients who are being considered for non-surgical palliative care. In this setting, senior multidisciplinary team members’ estimation should continue to be the major determinant in treatment choice. For the crucial six- to 12-month period, model performance is accurate enough to be a significant factor in the orthopaedic team’s decision to proceed with osteosynthesis or to undertake an endoprosthesis reconstruction, albeit considerations must be made about the model’s systemic pessimistic estimations.

Finally, validation of this and other prediction models should be a constant effort. The next logical step would be to analyse its performance in a multicentric cohort, to further validate its accuracy in the Portuguese population.
